# Linking genes to literature: text mining, information extraction, and retrieval applications for biology

**DOI:** 10.1186/gb-2008-9-s2-s8

**Published:** 2008-09-01

**Authors:** Martin Krallinger, Alfonso Valencia, Lynette Hirschman

**Affiliations:** 1 Structural Biology and BioComputing Programme, Spanish Nacional Cancer Research Centre (CNIO), C/Melchor F. Almagro, 3, E-28029 Madrid, Spain; 2 The MITRE Corporation, 202 Burlington Road, Bedford, Massachusetts, USA

## Abstract

Efficient access to information contained in online scientific literature collections is essential for life science research, playing a crucial role from the initial stage of experiment planning to the final interpretation and communication of the results. The biological literature also constitutes the main information source for manual literature curation used by expert-curated databases. Following the increasing popularity of web-based applications for analyzing biological data, new text-mining and information extraction strategies are being implemented. These systems exploit existing regularities in natural language to extract biologically relevant information from electronic texts automatically. The aim of the BioCreative challenge is to promote the development of such tools and to provide insight into their performance. This review presents a general introduction to the main characteristics and applications of currently available text-mining systems for life sciences in terms of the following: the type of biological information demands being addressed; the level of information granularity of both user queries and results; and the features and methods commonly exploited by these applications. The current trend in biomedical text mining points toward an increasing diversification in terms of application types and techniques, together with integration of domain-specific resources such as ontologies. Additional descriptions of some of the systems discussed here are available on the internet .

## Introduction

Life science research is characterized by the production of large and heterogeneous collections of biological data, including protein and genomic sequence data, expression profiles, and protein structure coordinates [1]. Although these data types represent an important fraction of existing biological information, they are often not amenable to direct human interpretation. A significant amount of information is encoded in the form of natural language, the main vehicle through which humans transmit and exchange information [2]. Most of the biological discoveries are communicated by means of scientific publications, patents, or reports, with an increasing number of them accessible via the worldwide web as electronic texts. Natural language is used to communicate information in a variety of other biological resources, including controlled vocabulary terms used for gene product annotations (for example, Gene Ontology [GO] terms) as well as in database records, such as in UniProt, which contain comments, keywords, or descriptions [3].

Structured database entries are designed to enable efficient data retrieval, exchange, and analysis. There has been a tendency to enrich annotation records of many of the existing expert-curated databases with previously missing but biologically relevant aspects by incorporating new fields or extending the inventory of terms used to describe these aspects. In parallel, new manually curated databases have been developed that cover a range of previously neglected biological entities, such as microRNAs or disease-associated single nucleotide polymorphisms; other new databases focus on highly specialized topics that are not sufficiently covered in general expert-curated databases [4]. Although the importance of more extensive annotations is becoming apparent, this is also associated with significant increase in curation workload, which slows down the manual annotation process. Even if general annotation databases such as UniProt - which stores a set of annotations linked to over 134,000 literature citations (February 2008) - are of great practical value, such databases are generally only capable of covering a small fraction of the biological context information that can be encountered in the literature. Crucial details of experimental conditions can still only be found in the underlying articles, making direct pointers to evidence passages from the literature especially important for interpreting existing annotations.

The bottom line is that biological databases alone cannot capture the richness of scientific information and argumentation contained in the literature; neither can databases provide support for the novel ways in which scientists will interrogate these databases. Even if curators of biological databases were able to keep up with the ever increasing volume of literature, biologists would still need text mining to link the database entries to the evidence and the argumentation contained in the literature.

The rapid accumulation of new publications, which must be processed by human curators for extraction of both new discoveries and revision of existing ones, represents an additional challenge for keeping biological databases up to date [5]. Traditional manual literature curation is only feasible for a small number of articles and a fraction of journals. The set of manual annotations derived from the literature also serves as the basis for so-called 'electronic annotations', in which functional information of curated entities is automatically transferred to other biological entities (without direct human curation) using computational sequence similarity methods [3].

Online literature collections such as PubMed, with over 70 million queries every month and over 17 million publications, are of crucial importance to both the experimental biologists and biomedical researchers, as well as to specialized users such as database curators. Centralized literature repositories like PubMed face double-exponential growth rates [6], which can be partially explained by inclusion of an increasing number of new journals, special issues or conference proceedings, and previously unindexed publications. Additionally, some journals have also augmented the number of articles included per issue, and - for those journals available only in electronic format - the only limitation in terms of number of accepted articles is the required effort involved in the peer-review process.

Efficient information retrieval (identification of relevant documents, given some search criteria) is essential to the biomedical research community [7], as large biomedical literature databases are being used as a resource for clinical decision support in evidence-based clinical practice, providing useful information for diagnostic aids [8]. Specific search strategies have been devised for optimal retrieval of relevant clinical studies to assist clinicians in performing efficient and targeted literature searches for specific medical subdomains [9,10]. Experimental biologists are making use of the scientific literature for multiple stages within the scientific discovery process. Knowledge extracted from previous publications is used to define the biological question or to select the actual target being studied, to extract information relevant for experimental set up (for example, biological conditions, parameters, and protocols), or to locate relevant resources (for instance, methodological systems or data repositories). After generating and analyzing the experimental results, information derived from the literature is essential to understand and interpret the resulting data, in order to draw conclusions about new discoveries. Finally, the results are communicated to the scientific community using publications in peer-reviewed journals.

For the pharmaceutical industry, text-mining systems are a valuable resource as part of drug discovery and target selection systems, but also for identifying adverse drug effect descriptions [11]. The pharmaceutical industry uses information technology applications to improve their competitive intelligence and knowledge management strategies, typically processing not just the scientific literature but also information contained in other textual data collections such as internal reports, patents, and newswire [12].

Modern biology is a dynamic, continually evolving research discipline, in which existing research topics and trends change rapidly over time [13]. For governmental institutions it is crucial to have a global view of the current research state and to monitor trends from the increasing number of scientific publications in order to ensure optimal resource allocation [14]. Publishers examine the literature to find domain experts for specific topics for the peer-review process and to ensure that their publications contain novel scientific discoveries, detecting potential cases of plagiarism. Tools such as Déjà vu, which uses text similarity calculations to detect duplicate citations from PubMed, can determine the novelty of publications [15]. The biomedical literature can also serve as a resource to build social networks of research collaborations using co-author citation analysis. Web-based applications such as PubNet are able to provide a graphical visualization of co-author networks derived from citations [16].

## Overview of current literature data resources

Several efforts have been undertaken to provide access to published medical and life science journal information, and make this information electronically accessible to the public through the worldwide web [17]. Most of these initiatives can be classified into one of three main categories.

1. Centralized institutional (for example, PubMed) or academic (for instance, Highwire Press and Hollis) repositories of peer-reviewed articles or article abstracts.

2. Article collection repositories hosted by publishers (for example, BioMed Central and EMBASE).

3. Online access to indexed scholar articles retrieved through web spiders and crawlers (for example, Google Scholar and Scirus).

A number of scholarly and scientific literature databases can be accessed online through search engines with simple query interfaces using keywords. Falagas and coworkers [17] compiled a list of literature databases containing summaries of articles of biomedical and life science journals with records both in English as well as in other languages. The most important resource for text mining applications is currently the PubMed database developed by the National Center for Biotechnology Information (NCBI) at the National Library of Medicine (NLM). Each of the citations contained in PubMed has a unique identifier (PMID) and can be accessed using Entrez, a text-based search and retrieval system. PubMed [18] includes citations (containing title, abstract, authors, and source information) submitted by participating publishers. Entrez improves the basic keyword searches by translating the user query to Medical Subject Heading (MeSH) terms, a hierarchical set of controlled vocabulary terms predominantly from the medical domain but also including terms for chemicals, genes, and proteins, used to index PubMed records [19]. In addition to basic searches using the Entrez retrieval system, PubMed also offers a more programmatic access to its content through the Entrez Programming Utilities [18] and popular open source projects such as the BioPerl, BioPython, and BioJava integrated libraries for retrieving PubMed content by biologist programmers [20]. The NCBI provides the My NCBI service [21] to periodically retrieve new publications in PubMed matching a predefined user query; the requester then receives a corresponding notification via an e-mail alert system.

To build a local relational database containing all PubMed citations [22], it is also possible to obtain a licensed copy of the whole of PubMed containing XML-formatted citation records from the NLM/NCBI. Some systems such as Txt2MEDLINE have even been implemented to allow access to PubMed using Short Message Service (SMS) queries, sending the users the results in text message format [23]; also PubMed Informer, a Web-based PubMed monitoring tool, facilitates PDA downloads and RSS feeds [24].

Alternative repositories and search engines to PubMed include Highwire Press and Google Scholar [25]. Google Scholar can recover not only peer-reviewed articles but also other scholarly texts, such as theses, books, and preprint repositories. A comparative study by Shultz [26] showed that Google Scholar often returns larger retrieval sets, but a substantial number are link-outs to PubMed records. Google Scholar currently also does not provide the advanced search functions offered by PubMed. HighWire Press (an initiative of Stanford University) represents another complementary resource to PubMed for accessing peer-reviewed articles, providing a search interface to over 1,160 journals and 4.8 million full-text articles, with over 1.9 million articles available free by Highwire partner publishers. A comparative evaluation of HighWire Press and PubMed in terms of search efficiency showed that although both share many search characteristics, they also have unique features [27]. Highwire Press has an option to provide a graphical visualization of the article's citation map and allows the user to further specify where to conduct the search (title, abstract, full text).

**Figure 1:**
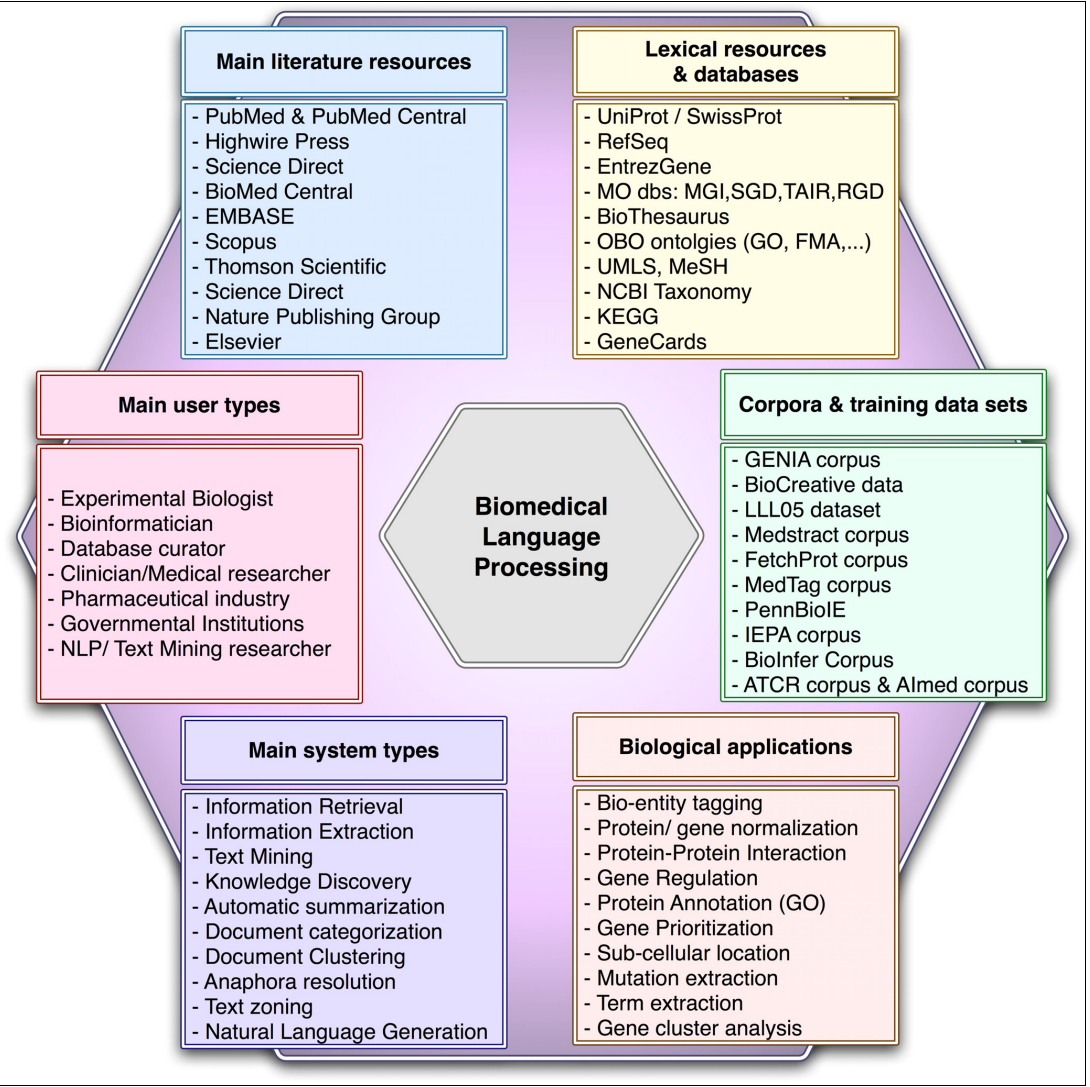
Overview of the main aspects relevant to the development of biomedical literature processing systems. ATCR, *Arabidopsis Thaliana *Circadian Rhythms; EMBASE, Excerpta Medica Database; FMA, Foundational Model of Anatomy; GENIA, GENome Information Acquisition; GO, Gene Ontology; IEPA, Interaction Extraction Performance Assessment; MGI, Mouse Genome Informatics; MO dbs, Model Organism databases; OBO, Open Biomedical Ontologies; RGD, Rat Genome Database; SGD, *Saccharomyces *Genome Database; TAIR, The *Arabidopsis *Information Resource.

Although article abstracts contain short descriptions that highlight the most relevant aspects of a given article, they only cover a small fraction of the information contained in full-text articles [28]. PubMed Central provides free online access to a electronic archive containing full-text articles of life sciences and biomedical journals. PubMed Central also contains articles published before 1966 that have been digitized as part of the Back Issue Digitization project [6]. Publishers have also developed platforms of searchable article repositories such as EMBASE or BioMed Central to improve the access to their articles. Figure 1 provides an overview of the main literature resources.

## The structure of biomedical language

The diversification process of protein sequences during the course of evolution is subjected to physical, chemical, structural, and historical constraints moulded by natural selection. Therefore, a collection of homologous protein sequences often shares a common structural fold and tends to exhibit a similar function. Similarly, in the case of natural language, a particular meaning may be expressed using different but largely synonymous expressions, also moulded by a set of structural constraints and historical events that shape languages, in this case English - the language of scientific communication. For example, the following three snippets of text below capture equivalent information about the interaction between the proteins VRK1 and c-JUN; these snippets illustrate some of the variations in word choice and syntax found in the scientific literature: 'VRK1 protein phosphorylates c-Jun' (example 1); 'the phosphorylation of c-Jun by VRK1' (example 2); and 'c-Jun is activated by VRK1' (example 3).

The techniques of natural language processing are used to 'decode' the information that is packaged in human language. This is done by exploiting the regularities and constraints that occur at multiple levels in human language. These levels include the following:

1. Words: lexical entries (words) are the units of meaning and the basic building blocks of language. A word is made up of a root and possibly other morphemes (prefixes or suffixes); for example, 'phosphorylates' in example 1 (above) consists of the root 'phosphorylate' plus the third person singular present tense morpheme '-s'. Morphemes can also modify the meaning or change part of speech; for example, the suffix '-tion' changes the verb ('phosphorylate') into the related noun ('phosphorylation'), and the prefix 'de-' can negate the meaning, as in 'deactivation'.

2. Syntax: the syntax or grammar of a language controls how words are grouped into meaningful phrases and eventually into sentences. For example, in English, word order is used to convey grammatical relations such as subject-verb-object. In example 1 (above), the noun 'VRK-1' is the subject (and actor) for the verb 'phosphorylate' in the sentence 'VRK1 phosphorylates c-Jun', whereas 'c-Jun' is the object (recipient of the action). To aid in syntactic analysis, words can be associated with part-of-speech (POS) tags, to distinguish nouns, verbs, prepositions, and conjunctions. To link words to their grammatical function, each word can be assigned a 'part of speech', indicating its role in the sentence, for instance a noun (name of something), verb (an action or linking word), an adjective (describing a quality), and so on. POS taggers are computer programs that automatically assign each word its corresponding POS label. These systems are generally based on machine learning algorithms such as hidden Markov models trained on manually POS-labeled text collections (corpora). The biomedical literature shows a slightly different POS distribution as compared to general English newswire texts, which has motivated the implementation of specialized taggers optimized for the biomedical domain, such as the MedPost [29] tagger or dTagger [30]. POS information can be useful to detect textual patterns expressing protein interactions [31] or to locate gene and protein mentions [32].

3. Semantics: semantic relations capture meaning; for instance, example 3 (above) 'c-Jun is activated by VRK1' can be represented as an operator (the verb 'activate) operating on two arguments - 'activate (VRK1, c-Jun)' - in the same way that a logical operator operates on its arguments. The semantic representation abstracts away from the details of the underlying syntax (and specific words), to capture regularities. Thus, in this example, the semantics capture the fact that VRK1 does the activation, and c-Jun is activated.

4. Pragmatics: pragmatic or discourse relations capture the larger context and its contribution to meaning. Text-mining tools often rely on sentences as the basic processing unit for extracting associations between biological entities. However, descriptions of these relations go beyond sentence boundaries and make use of referring expressions [33], as is the case in the following two sentences from (PMID 15800059): (a) '*Dictyostelium LIS1 *(*DdLIS1*) is a microtubule-associated protein exhibiting 53% identity to human LIS1.' (b) '*It *colocalizes with dynein at isolated, microtubule-free centrosomes, suggesting that both are integral centrosomal components.'

**Figure 2:**
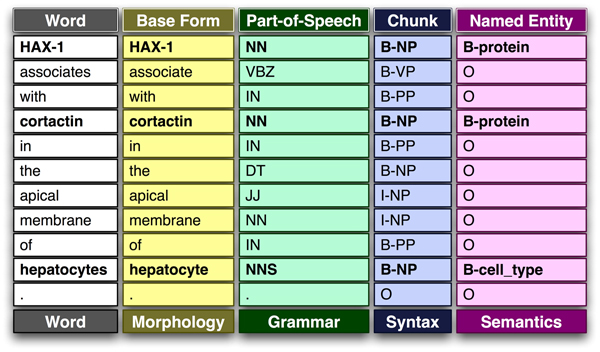
Main natural language processing levels, from word tokenization to semantics. The different processing layers for a given example sentence are shown here. This example is based on the output generated by the GENIA tagger: DT, determiner; IN, preposition or subordinating conjunction; JJ, adjective; NN, Noun (singular or mass); NNS, Noun (plural); VBZ, Verb (third person singular present). The B/I/O terminology refers to begin phrase (B), internal to phrase (I), and outside of phrase (O).

These layers of structure provide constraints, reduce ambiguity, limit redundancy, and enable efficient communication (Figure 2 provides an example case illustrating these different levels of language complexity). Also, much in the same way that genomic sequences can be 'parsed' to identify specific patterns such as genes, control regions, or - on a larger scale - motifs, linguistic structure leads to regularities that can be exploited by automatic text processing systems to learn the statistical properties of human language and to decode the information it contains - often using the same kinds of pattern recognition techniques that are used to analyze genomes and proteomes.

The presentation of biological information in the literature interacts with the general linguistic structures described above, but is also subject to peculiarities associated with the specific domain (here, biomedicine). This specialized usage of language in a domain is known as a sublanguage. The sublanguage(s) of science have been extensively studied by linguists [34-36].

The understanding of sublanguage structure underlies much of current text mining and natural language processing, as is discussed in more detail in the next section. The literature of a particular subfield (for example, molecular biology) has characteristic terms (for instance, 'gene', 'protein', and 'phosphorylation') and characteristic collocations (co-occurrences of terms used as phrases, such as 'cell membrane' or 'ion channel'). Techniques for document search and clustering make heavy use of profiles based on the distribution of single words and multi-word phrases to find and rank documents relevant to a particular search, as was required for the BioCreative protein-protein interaction task ('find all articles with experimental evidence for protein-protein interaction'). In addition, the sentence-level patterns of word occurrence are used by entity recognition and entity normalization systems to identify 'names' of types of entities, for example genes, proteins, or species. These systems make use of the word and sentence context to detect kinds of entities; however, these contexts are quite specific to the particular subfield and may well evolve with time, as new experimental methods come into use and new kinds of entities are discovered. Therefore, the sublanguage associated with a subfield evolves over time and must be constantly updated. In addition, another contributing factor derives from the fact that English is the language of scientific publication, but many authors have different native languages that may influence their writing style, including sentence length and word usage; this leads to greater variability in these aspects when compared with texts written by native English authors [37].

Like any subfield, biomedicine makes heavy use of domain-specific terminology and relies also on typographic and orthographic conventions to communicate certain kinds of information. This in turn affects 'tokenization', or the process of identifying the strings of characters that make up words. Fortunately, there are rich resources for biomedical terminology that can be used to build lexicons, to create linkages from one set of resources to another, and to learn associations, for example ontologies such as GO. These three topics are discussed in the following subsections.

### Biological terminology

The biological literature is characterized by heavy use of domain-specific terminology. There are estimates that more than 12% of words found in biochemistry publications correspond to technical terms of that scientific discipline [38]. This has motivated the development of strategies to recognize biomedical terms and their variations automatically [39].

There are two basic challenges in dealing with terminology; the first is the constant formation of new terms and new 'short forms' or abbreviations. This is related to the second problem of ambiguity or polysemy (multiple meanings of a word). Ambiguity results when an existing term is used to describe a new concept (for instance, a new gene or protein), or when a new abbreviation is coined that turns out to be identical to another abbreviation. Selection of the correct meaning of a polysemic word requires understanding of the context of occurrence. For example, in the sentence 'The *Drosophila *peanut gene is required for cytokinesis and encodes a protein similar to yeast putative bud neck filament proteins' (PMID 8181057), 'peanut' corresponds to a fly gene name, whereas in 'Peanut (*Arachis hypogaea*) forms root nodules in a unique process.' (PMID 18256023) it corresponds to the name of a plant. Text-mining tools must be able to select the correct sense of a word dependent on its context (word sense disambiguation). Disambiguation is particularly important in correctly associating genes mentioned in the literature with their corresponding database entries. Gene names are a problem because they are often shared across species, especially between mouse and human. Chen and colleagues [40] showed that general English words had a relative low ambiguity (0.57%) when compared with the greater ambiguity of medical terms (1.01%) or the much greater ambiguity among gene names (14.20%).

The biomedical and life science literature also relies on heavy use of short forms (acronyms or abbreviations), leading to further ambiguity [41]. For instance, the acronym APC can correspond to one of the following expanded forms, depending on its context: antigen-presenting cells, adenomatous polyposis coli, activated protein C, anaphase-promoting complex, and argon plasma coagulation (based on the output of Acromine [42]). Accessible online search tools for acronym-full name pairs include ADAM [43], the Abbreviation Sever [44], and AcroMine [42].

As new biological discoveries are made, new experimental methods are developed and novel gene names (or their synonyms) and functional terms are created [45]. Thus, existing lexical resources and annotation databases must be constantly updated to integrate this new information. To add to the problem, biologists often do not adhere to naming standards [46]. As a result, simple dictionary look-up based techniques are not effective in detecting novel names that are not yet contained in a database; therefore, these names can not be directly identified by pattern matching, but must be extracted based on contextual information, such as occurrence before the word 'gene', or because the term precedes the phrase '... is transcribed'.

### Tokenization and morphology: identifying words in biological text

The first step in processing any text requires segmentation of the string of characters into words. Normally, word boundaries (in English) are indicated by white space, and a sentence boundary is indicated by '.' (period or full stop). However, there are many complications, particularly in the scientific literature; examples include use of '.' in decimals ('1.09'), use of '/' to link multiple gene names ('waf/cip-1'), or variable use of white space in gene names, such as 'BRCA 2' versus 'BRCA-2'. Tokenization typically requires typographical processing at the character level, to handle special characters and white space, upper case and lower case, superscripts and subscripts, and equivalence of Roman, Greek, and numerical suffixes ('TNFa' versus 'TNF-alpha'). For example, in order to retrieve all of the mentions of the BRCA2 gene in the literature, a gene mention retrieval system would need to capture at least the following typographical variants: Brca2, Brca-2, BRCA-2, and BRCA 2. To improve tokenization of life science articles, the JULIE (Jena University Language and Information Engineering) laboratory provides tools that can be used for detecting token and sentence boundaries [47].

This stage of processing is very important, particularly for systems that identify gene mentions and link these mentions to specific entries in biological resources (gene normalization); see [48] (especially Table 2 in that report) for a list of techniques and resources used in the systems participating in the BioCreative II gene mention task. Some of the teams that participated in BioCreative II [49] explored the integration and use of publicly available gene mention taggers, such as the ABNER application [50] or the LingPipe system [51]. At the data preprocessing stage, token segmentation also plays a role in correctly normalizing (linking) gene mentions to database records, as was shown by participants of the BioCreative II gene normalization task [52].

The tokenization process is related to morphological analysis of the internal structure of the words. For example, the use of suffixes to detect protein mentions in the literature has been studied in detail [32]. The process of 'stemming' can be viewed as a kind of (impoverished) morphological processing, which maps words into their 'stems', thus reducing variability and providing better clusters. The intuition is that the meaning is carried predominantly by the stem or root, and therefore it is appropriate to collapse variants into a single class. Stemming is heavily used in document retrieval and clustering applications for building models of word distribution across document collections. Most current applications use general stemming strategies such as the Porter Stemmer algorithm [53], but also some recent efforts rely on specific biomedical stemmers [54].

### Lexical and semantic resources for biology

Functional descriptions of bio-entities, relevant biological processes, or experimental techniques are often expressed in scientific papers using domain-specific technical terms. Terminological repositories and dictionaries are important resources to assist in the interpretation of scientific articles, but also for building biomedical ontologies used for extracting biological annotations and to assist authors in consistent use of domain specific terminology.

Fortunately, as part of the development of biological databases, biologists and database curators have made available important resources cataloging and organizing the terms and their synonyms used in these areas. In addition, there are an increasing number of ontologies being developed for various fields and subfields of biology, particularly the GO [55]. Biomedical ontologies, and especially GO, are widely used as controlled vocabulary to describe biologically relevant aspects of gene products. Although GO was primarily designed for annotation purposes, it can also be used as a lexical resource for indexing and navigating the biomedical literature through the underlying network of concepts using the GoPubMed application [52,56]. The GOAnnotator tool [57] allows extraction of text-based GO annotations for a given protein identifier (SwissProt accession number) by automatically mapping all of the protein names contained in the corresponding SwissProt record to PubMed abstracts. These abstracts are then associated with GO terms based on text similarity between the term and abstracts, using the GO hierarchy to improve the overall precision [58]. The GO annotation assignment task of BioCreative I showed that, in general, the automatic detection of GO terms was more efficient in case of short terms, and especially for terms corresponding to the cellular component category [59].

In addition, as seen in both the first BioCreative and this recent BioCreative, expert curated databases provide important 'gold standard' datasets that can be used in formal evaluations, such as BioCreative, or to explore new tasks. Automatically linking information from life science literature into structured representations was pioneered by Mark Craven [60].

At the word level, valuable resources include gene/protein name dictionaries compiled from biological annotation databases such as SwissProt, as well as domain-specific ontologies (for example, GO) and thesauri. For example, BioThesaurus is a widely used resource [61] that has compiled gene and protein names from multiple sources. Such resources can be used, for example, by dictionary look-up based strategies to link articles to protein database records automatically [62], or to develop controlled vocabularies of functional terms [56]. However, as discussed above, existing resources may not cover all the genes and proteins and their many variations and abbreviations; in addition, the more complete the resource, the more likely it is to contain terms that have multiple meanings. For terminology development, the TerMine system developed at the National Centre for Text Mining (NaCTeM) integrates an automatic term recognition approach using linguistic and statistical analysis of candidate terms [63].

## Biomedical literature processing applications

Biomedical literature processing tools provide access to information contained in scientific articles at various levels of granularity, both in terms of the queries supported as well as in terms of the results. We describe the building blocks for biomedical text processing with reference to the general BioCreative tasks:

• Document retrieval, which is the core of the 'interaction article' subtask, to select articles about protein-protein interactions.

• Entity mention, which requires identification of mentions of biological entities (for BioCreative I and II, specifically genes or proteins) in text.

• Entity normalization, which links biological entities, such as genes or proteins, to biological resources, such as SWISS-PROT or Entrez Gene.

### Document retrieval

Document retrieval requires the ability to process and index massive volumes of data (for instance, the entire MEDLINE collection). This means that techniques used to index the collection must be robust and efficient with respect to space and time. For biomedical processing, the most obvious approximation [64] is to look for keywords that characterize a collection of papers, based on keyword frequency. This system forms the basis of neighbor searches in MEDLINE, which is the predecessor of eT-Blast and still the most heavily used system. Many subsequent approaches have used alternative strategies for collecting papers and obtaining statistics for words or terms, with or without context.

Many current literature mining approaches rely on statistical analysis of word occurrences, calculated over the whole PubMed database and resulting in weighted associations between biological entities. The underlying assumption of these global strategies is that if two biological entities frequently co-occur together or appear in similar contexts (documents), then they should have some biological relationship. These methods can provide 'high recall' systems that return large numbers of possibly relevant documents. Such datasets can be refined by use of more sophisticated (and time consuming) processors such as relation extraction systems that examine single sentences. Such systems have the potential to capture multi-document based relations that are currently missing in curated biological databases.

Statistical co-occurrence based relation extraction poses a challenge in terms of human interpretation, because it lacks semantic information on the type of biological association. Systems such as the CoPub Mapper [65] provide online access to ranked co-occurrence associations extracted from PubMed between genes and biological terms (for instance, from GO or disease names) and the PubGene system generates a graphical protein interaction network based on protein-protein literature co-occurrences [66].

Stemming algorithms convert words into standardized forms (stems) and are an essential component of information retrieval systems and search engines [67]. One common shortcoming when using stemming algorithms is that they sometimes collapse two semantically different words to a common stem. Stemming has been used by systems to quantify the similarity between documents (for example, eTBlast [68]) and by document categorization [69] or document clustering [70] approaches.

At the document level, text processing applications like CoPub [65] detect over-represented terms from multi-abstract collections, a strategy which uses automatically generated document-gene links to provide biological context, to assist in the interpretation of sets of genes resulting from large scale experiments, such as gene expression microarrays. Text similarity algorithms have been integrated into eTBlast [68], an online application ranking the retrieved PubMed records according to their similarity to a given input query article. This kind of system represents a useful strategy for authors to improve retrieval of relevant references when writing a scientific publication, as well as a practical system for publishers to avoid plagiarism. Clustering algorithms have been used to group genes according to their expression profiles in microarray experiments or to build phylogenetic trees by examining similarities of biological sequences. For protein sequences, a common strategy for measuring similarity uses weighting of amino acids based on their substitution rates [71]; similarly, for calculating document similarity, terms are often weighted according the number of times a term occurs in a given document (term frequency using local or within-document information) and by the number of documents containing this term (document frequency, using global, or within-collection information) [72].

Document clustering approaches using document similarity calculations have been used by PubClust [73] and McSyBi [74] to structure further the collection of articles retrieved by keyword searches. A recurrent challenge in both bioinformatics and text processing is the classification of a collection of items into a set of predefined categories. The assignment of labels to whole documents, sentences, or individual word tokens given their context (sentence) has been addressed using machine learning techniques for cases where suitable manually labeled training text collections have been constructed. Some published systems use document classification strategies to detect whether a given article describes biologically relevant aspects, such as protein interactions, subcellular location information [75], or even enzyme kinetic parameters [76].

For humans it is often more effective to retrieve specific descriptive sentences or passages rather than to look at whole documents or abstracts. Also, for information extraction tools extracting relations between biological entities, the detection of sentences potentially containing these associations can improve performance [77]. Therefore, specific sentence classification strategies have been proposed both for genetic interactions [77] and for protein interactions [33].

### Gene mention and gene normalization

Biologists often search annotation databases using gene/protein names or symbols as queries. The names currently stored in such resources have been manually extracted from the literature, a time-consuming task that generally is unable to cover all of the synonyms or naming variants used by biologists for each gene and mentioned in the literature. Automatic detection of protein and gene mentions from the literature is not only useful to improve coverage of annotation databases or to enable a semantically refined literature search, but also constitutes a crucial initial step for other text-mining applications that extract relations or properties of these biological entities. Detection of gene mentions is the focus of the BioCreative gene mention task; see [78] for a summary of the approaches used in BioCreative II. The performance of gene mention systems has increased from the first BioCreative, and when multiple systems are combined the combined BioCreative II systems have achieved an estimated F measure (harmonic mean of precision and recall) of over 90% (see [78]). Because most entity mention tagging systems rely heavily on machine learning and statistical methods, they have benefited greatly from availability of large quantities of training and test data. For BioCreative II, there were 15,000 training sentences and 5,000 blind test sentences.

Krallinger and Valencia [79] provide an overview of current systems and discuss the main difficulties encountered by gene mention detection systems. Most of the current bio-entity recognition systems, like GAPSCORE [80] or ABGENE [32], can label text for protein or gene mentions; other applications, such as ABNER [50], also identify cell lines or cell types. Other biological entities of interest include chemical compound mentions, a crucial component for systems trying to extract biological pathways, and enzyme-ligand associations. Oscar is an open source system for recognizing chemical entity mentions; it integrates a dictionary of compound names, as well as using regular expressions, heuristics, and certain word combinations to find chemical names in text [81].

Finding mentions of species and taxonomic names is not only important for the emerging field of biodiversity informatics [82], but constitutes a crucial step in linking gene mentions to their corresponding organism source. Therefore, systems such as TaxonFinder (uBioRSS) [83] or TaxonGrab [84] that tag taxonomic names in electronic literature can provide improved access and integration of species-specific information contained in publications.

The detection of bio-entity mentions alone is often not enough to retrieve informative sentences efficiently, especially when the resulting document collection is of considerable size. The BioIE system tries to detect, for a given query keyword, only those sentences describing aspects related to protein families, functions, structural characteristics as well as associations to diseases [85]. Other applications such as iHOP map a given gene or protein query name to its corresponding database identifier and then retrieve a collection of sentences with definition information, highlighting co-occurring MeSH terms [86]. An alternative to providing term co-occurrence sentences is offered by EBIMed and FACTA, which - for a given query protein - present a summary table of co-occurring concepts based on PubMed abstracts. These concepts include other proteins, GO terms, drugs, and species mentions for EBIMed [87], and proteins, diseases, symptom, drugs, and compounds in the case of FACTA. FABLE allows retrieval of all the co-occurring gene and protein mentions for a query keyword, applying a context-based disambiguation strategy to determine whether a possible mention corresponds to a gene or not (also expanding gene searches with its corresponding synonyms). Results retrieved from FABLE can be downloaded in several formats, including XML and Excel.

**Figure 3:**
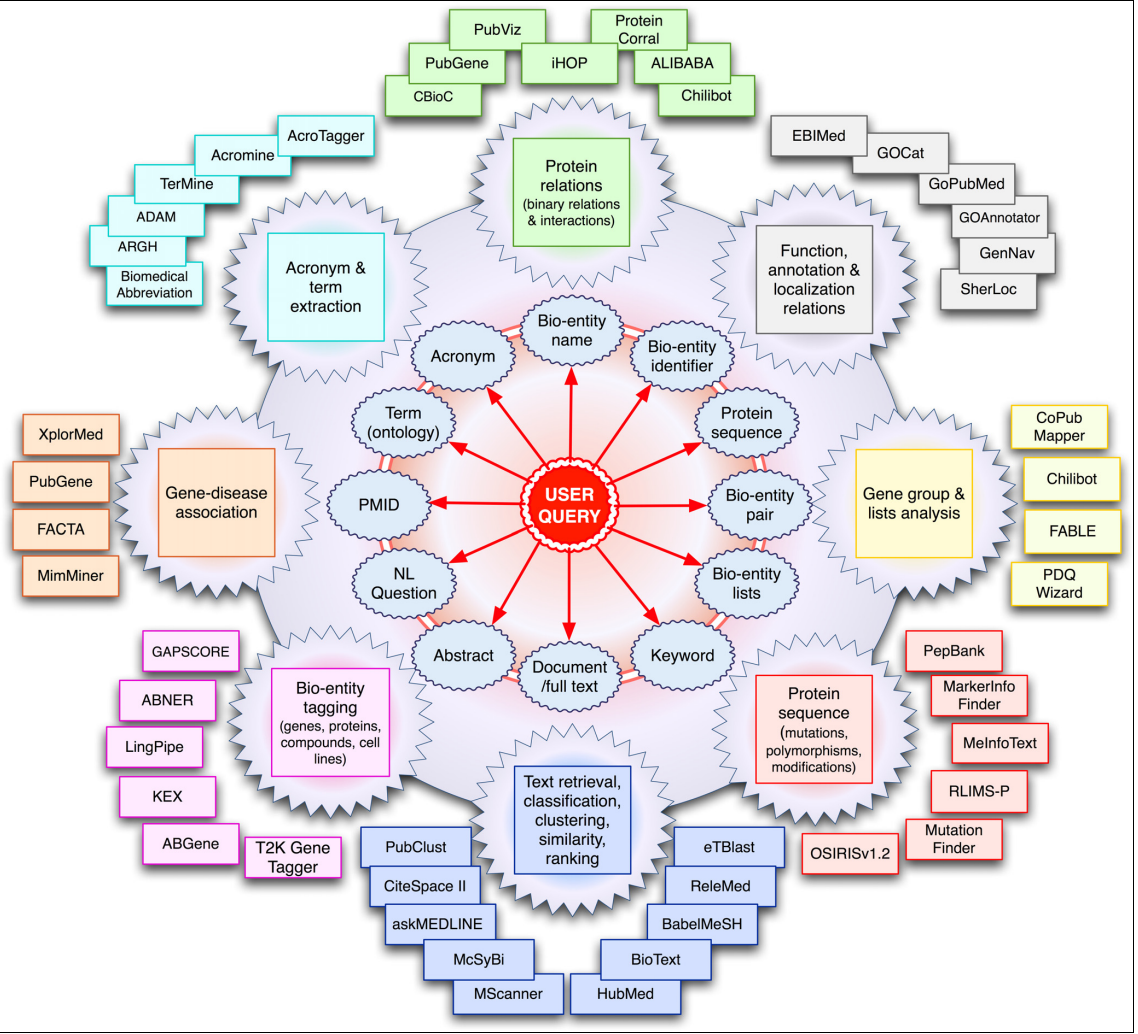
Biomedical text mining applications from the biology user perspective. This figure provides a simplified general overview of some existing biomedical text mining applications from the biology user perspective. The main user query types currently addressed by existing literature processing applications are shown in the center of this figure. The outer circles represent the type of implemented applications as well as some of the corresponding systems. Note that some tools could in principle be associated to several application types (but only one of them is illustrated here). For a more detailed description of the displayed systems refer to the online tool collection repository.

When searching the literature for functional information for gene products, it is not necessary to use gene names as queries. Instead of using gene names, searches can be conducted using protein sequences through the METIS [88] or the MedBlast [89] systems. They integrate the intermediate step of sequence similarity searches to link the query sequence to its corresponding database record and then automatically retrieve the associated literature, exploiting the corresponding gene names and citations provided in the database records. Figure 3 provides an overview of the main user query types and text mining applications relevant to the biomedical domain.

## Beyond BioCreative: advanced applications

The extraction of biological relations between bio-entities can provide insights into their functional characteristics. Attempts have been made by Rodriguez-Penagos and colleagues to automatically extract genetic interactions from abstracts and full-text articles, in order to improve efficiency of the manual curation effort of transcriptional regulatory networks in the *Escherichia coli *database RegulonDB [90].

Other systems such as iHOP and InfoPubMed allow retrieval of protein interaction sentences from PubMed. iHOP links the interacting proteins to their corresponding database records and allows navigation in the resulting network of co-occurring interaction proteins as well as building a graphical interaction network. In the case of InfoPubMed, first an interaction summary is generated for a given query protein and then, by dragging the selected interaction pair into a content viewer, the evidence sentences are displayed. For finding supporting relationship evidence between two predefined entities of interest (genes, proteins, keywords), or even between two lists of entities, the Chilibot application [91] can be used. It also generates a graphical network summarizing the relationships, providing qualifiers for the type of relation (stimulative, inhibitory, neutral).

Recent text-mining applications have tackled the extraction of specific biological attributes of genes or proteins, such as their sequences, polymorphisms and mutations, residue modifications (for example, phosphorylation), or even their subcellular locations. MutationFinder [92], a rule-based system, can extract amino acid mutation mentions from large text collections. Other approaches like MarkerInfoFinder try to detect information related to sequence variants of human genes, such as single nucleotide polymorphisms and other types of genetic markers, and their association with diseases [93]. To develop the PepBank database [94] containing a collection of peptide sequences, a text-mining system was used that automatically detected and extracted peptide sequence mentions from abstracts and full-text papers. The Phospho.ELM database [95] integrated a text-mining system in order to automatically detect S/T/Y phosphorylation sites from the literature.

Increasing interest in studying epigenetic modifications of the human genome and its association with diseases such as cancer motivated the development of two other online tools, namely MeInfoText [96] and PubMeth [97], which use text-mining to provide detailed information on gene methylation and association with cancer. Knowledge about the subcellular location of proteins can provide meaningful contextual information about potential interaction partners or protein function. The EpiLoc system [75] constitutes a text-based subcellular location prediction tool, effectively complementing alternative sequence-based localization prediction algorithms.

Mining the literature also offers an opportunity to extract indirect associations or discover new relationships based on the analysis of multi-document collections. High-throughput experimental setups often result in large lists of candidate genes that must be experimentally characterized in more detail. To rank (prioritize) genes according to some co-occurring user-defined keyword, the PDQ Wizard [98] allows interactive filtering of results and display of publication information to customize the ranking strategy. The literature-based discovery system ARROWSMITH [99] supports extraction of indirect relationships between two different topics or keywords by examining the commonalities (shared words and phrases) between the two article collections mentioning to each topic.

## Conclusion

Existing biomedical literature processing applications cover retrieval, ranking, or clustering of relevant articles for a particular topic or bio-entity. There are tools developed for the extraction of biological associations such as protein interactions, gene regulation, or functional annotations, as well as detection of biologically relevant properties for genes and proteins such as sequence mutations or gene methylation information. A collection of application descriptions and links to the corresponding online systems, together with relevant references, can be found online [100]. To be of practical value for the life science community, developers of text mining applications need to keep in mind some critical issues, listed below.

### Linking literature to experimental results

Linking text directly to unique database identifiers or sequences is crucial, especially because protein and gene names are often ambiguous. Modern biology is characterized by experimental studies examining large collections of genes or proteins; therefore, text-mining systems should support retrieval of relevant information from the literature for gene/protein lists, not just single gene queries. For experimental sciences such as molecular biology, efficient access to experimental information is crucial. Thus, automatic extraction of evidence qualifiers is crucial when automatically extracting protein interaction relations or annotations of gene products with functional concepts (for example, GO terms) from the literature. Aspects of interest include experimental techniques supporting these relations, type of experimental set up (*in vivo*/*in vitro*), and relevant contextual information such as cell lines, tissues, or model organism systems.

### Linking text mining to bioinformatics resources

Bioinformatics applications and biological databases frequently provide external references to other resources or tools, which improve data integration and allow, for example, navigation from functional annotation databases to protein family or structure information. Biomedical literature processing tools should improve both the connection to other literature mining systems (for example, through meta-systems) as well as to existing biological annotation resources and bioinformatics applications. Integration of additional data types such as figures and also specific processing of tables and references from full-text articles, patents, and e-books will gain importance in the future because of the increasing number of electronically available texts stored in open access repositories. To allow robust integration of different systems, standards for commonly accepted text annotation formats and use of controlled vocabularies and ontologies are essential aspects.

### Accessibility, flexibility, update, and maintenance of literature mining systems

Journal publications provide useful pointers to resources and software, but usually only a subset of the corresponding URLs are links to stable systems [101]. To be of practical value to both users of the biology community and to text-mining developers, there must be not only a detailed description of algorithms and text-mining methods for a system, but also a stable implementation of the corresponding system. Ideally, the implementation of any published application should be accessible through multiple strategies: as an online system, enabling flexible navigation and visualization of extracted information, with facilities for saving and exporting results in various formats; as a web service for a more programmatic access, providing predictions in standard formats such as XML; and even as a package that can be installed locally and customized for other specialized systems. Considering the accelerating pace of new discoveries in molecular biology reported in scientific articles, it is also crucial that text-mining systems be systematically maintained and updated, periodically including new publications. Literature processing tools must be efficient enough to scale up, processing the entire PubMed database as well as large collections of full-text articles available in a range of different formats.

### Comparative evaluation and user interaction

To determine the performance of literature processing tools, meaningful system evaluations and comparative studies with other methods are necessary. Initiatives such as the BioCreative challenges provide the opportunity for text-mining developers to participate in independent community assessment studies, which are especially important, given the difficulty in constructing suitable evaluation datasets. Current systems could benefit from formal characterizations of the main end user types, detecting their specific needs and allowing user interaction and feedback to be taken into account for iterative improvement of system usability. Efficient ranking and reliability scoring of results are helpful to improve retrieval of the desired information, reducing the workload in terms of manual examination of the text mining output. Accurate documentation and clear examples of what a given system can actually do and what it should be used for can bring literature processing strategies closer to the end users. Most of the biological literature is currently published in English, but when trying to bridge clinically relevant aspects, there is a clear need for cross-language information extraction applications to access articles published in other languages with functional descriptions of biological entities.

### Future challenges: personalized text mining and text-mining workflows

Literature processing tools can go beyond the single document-based biological annotations that are currently stored in biological databases by exploring the global collection of available papers. Nevertheless, it remains difficult for humans to interpret weighted relations based on multi-document collections. One of the potential sources of errors from multi-document derived associations is related to incorrect linking of articles to biological entities (for example, grouping papers corresponding to different proteins that share the same name or abbreviation).

Biologists and database curators often carry out repetitive multi-step literature searches, using the output generated by one literature search as input for the next one. Text-mining workflows inspired by manual literature searches and curation pipelines might be useful in the future, but only if automatically generated text-based outputs are accurate enough to produce meaningful results. With the increasing specialization in molecular biology research and the pressing need to keep up with new scientific discoveries, there is also a clear need for personalized literature recommender systems and text-mining systems that can provide for each scientist the information that he or she is particularly interested in [102]. Systems such as Mscanner, which classify the literature based on a collection of user defined PubMed articles, constitute a step in this direction [103].

## Abbreviations

GO, Gene Ontology; MeSH, Medical Subject Heading; NCBI, National Center for Biotechnology Information; NLM, National Library of Medicine POS, part-of-speech.

## Competing interests

The authors declare that they have no competing interests.

## Authors' contributions

MK was responsible for authoring the manuscript draft. LH provided material on the linguistic structures. AV and LH supervised, revised, and edited the article.
